# The causal impact of saturated fatty acids on rheumatoid arthritis: a bidirectional Mendelian randomisation study

**DOI:** 10.3389/fnut.2024.1337256

**Published:** 2024-02-12

**Authors:** Xiaoling Yao, Yuzheng Yang, Zong Jiang, Wukai Ma, Xueming Yao

**Affiliations:** ^1^Second Clinical Medical College, Guizhou University of Traditional Chinese Medicine, Guiyang, China; ^2^Department of Rheumatology and Immunology, The Second Affiliated Hospital of Guizhou University of Traditional Chinese Medicine, Guiyang, China

**Keywords:** rheumatoid arthritis, saturated fatty acids, mendelian randomisation, causality, bidirectional

## Abstract

**Objective:**

The causal relationship between saturated fatty acids (SFAs) and rheumatoid arthritis (RA) remains poorly understood. This study aimed to determine whether SFAs are causally related to RA using Mendelian randomisation (MR) analyses.

**Methods:**

Genome-wide association study (GWAS) summary data for RA (ukb-d-M13_RHEUMA) and SFAs (met-d-SFA) were obtained from the Integrative Epidemiology Unit OpenGWAS database. A bidirectional MR analysis was performed using a suite of algorithms, namely the MR-Egger, weighted median, simple mode, weighted mode, and inverse-variance weighted (IVW) algorithms, all integrated using the “MR” function. The robustness of the MR findings was further evaluated through sensitivity analyses, including heterogeneity, horizontal pleiotropy, and leave-one-out tests.

**Results:**

The IVW algorithm in the forward MR analysis indicated a causal link between SFAs and RA (*p* = 0.025), identifying SFAs as a risk factor for RA (odds ratio = 1.001). Sensitivity analyses indicated no significant heterogeneity, horizontal pleiotropy, or severe bias, reinforcing the credibility of the forward MR results. However, the reverse MR analysis revealed that RA does not causally affect SFA levels (*p* = 0.195), and this finding was supported by corresponding sensitivity analyses.

**Conclusion:**

The findings of this study substantiate the positive causal effect of SFAs on the incidence of RA through bidirectional MR analysis, thereby offering a consequential direction for future research on the diagnosis and treatment of RA.

## Highlights

- The causal relationship between SFAs and RA was evaluated in a two-sample bidirectional MR analysis.
- SFA was causally related to RA as a risk factor, with an increase in SFA levels leading to an elevated risk of RA.
- RA was found to have no causal effect on SFA.

## 1 Introduction

Rheumatoid arthritis (RA) is a complex chronic autoimmune disorder with an uncertain aetiology and is characterised by persistent inflammation resulting in synovitis, pannus formation, and gradual degeneration of articular cartilage and bone. Symptoms typically include joint swelling, deformity, and muscle atrophy, leading to functional impairment (1, 2). The 2017 Global Burden of Disease study indicated rising age-standardised prevalence and incidence rates of RA from 1990 to 2017 (3). The lack of targeted therapeutic solutions for RA necessitates a deeper understanding of its aetiological factors to develop strategies for slowing its progression.

Various factors have been implicated in RA pathogenesis, such as genetic predisposition, environmental exposures, metabolic disturbances, autoimmune responses, and microbial influences (4–11). Fatty acids (FAs), which are ubiquitous in human tissues, play vital roles in tissue homeostasis, immune function modulation, and metabolic pathways (12, 13). Whilst the role of unsaturated fatty acids in RA has been the focus of recent research, the impact of saturated fatty acids (SFAs) remains relatively unexplored (14, 15). SFAs that lack double bonds in their carbon chains are essential lipid components (16). A recent observational study suggested that excessive SFA intake might trigger inflammation and muscle degradation in patients with RA, possibly leading to sarcopenia and inflammatory processes (17). The American College of Rheumatology dietary guidelines for RA recommend a Mediterranean diet with limited SFA intake (18). Nevertheless, given the extant controversies and inherent biases in observational research methodologies, it is imperative to rigorously assess the causative implications of SFAs for RA.

Observational studies have found an association between SFAs and RA. However, neither the direction nor the cause–effect chain is clear. By leveraging inherent genetic variations, Mendelian randomisation (MR) serves as a robust statistical approach to assess causality between SFA and RA (19, 20). MR is superior to traditional observational methods in controlling confounders and minimising potential biases from confounding and reverse causation, and it can thus provide more reliable evidence for establishing causality between SFA and RA (21). This study aimed to investigate the causal relationship between SFA and RA through a two-sample bidirectional MR analysis, contributing to future aetiological research on RA.

## 2 Materials and methods

### 2.1 Data sources and summary

The genome-wide association study (GWAS) summary data on RA and SFAs were retrieved from the Integrative Epidemiology Unit OpenGWAS database.^1^ The data on RA (ukb-d-M13_RHEUMA) were obtained from 1,605 cases and 359,589 controls and covered 10,079,899 single-nucleotide polymorphisms (SNPs). This dataset was sourced using the keyword “Rheumatoid arthritis” and selected from the UK Biobank database^2^ results. For SFAs (met-d-SFA), the sample and SNP counts were 114,999 and 12,321,875, respectively.

### 2.2 GWAS data pre-processing

The “extract_instruments” function of the R package “TwoSampleMR” was adopted to read the data on exposure factors and filter the instrumental variables (IVs) (22). SNPs significantly associated with exposure factors were selected as IVs (*p* < 5 × 10^−8^), and those in linkage disequilibrium were excluded (clump = TRUE, *r*^2^ = 0.001, kb = 10,000). Each SNP was also cross-referenced with the PhenoScanner GWAS database to check for associations with vitamin D, arthrosis, and pain as potential confounding factors. These SNPs were not associated with the confounding factors of RA. Simultaneously, the SNPs that were markedly related to the outcome, identified based on GWAS data on the outcome, were also removed. In the forward MR analysis, the exposure factor was SFA, and the outcome was RA. In the reverse MR analysis, SFAs and RA were interchanged as the outcome and exposure factors, respectively. The F-statistics for each genetic instrument were assessed to ensure method reliability. An *F*-value of >10 indicated a low likelihood of bias due to weak instruments.

### 2.3 Bidirectional MR analysis

The MR analysis was based on three core assumptions for the IVs: (1) genetic variation is associated with risk factors; (2) genetic variation is not associated with confounding factors; and (3) genetic variation affects the outcome solely through risk factors (23). First, the “harmonise_data” function was employed to harmonise the effect equipotential with the effect size. The “MR” function of the R package “TwoSampleMR” and five algorithms, namely MR-Egger, weighted median, inverse-variance weighted (IVW), simple mode, and weighted mode, were used to perform the bidirectional MR analysis. The MR results primarily relied on the IVW algorithm due to its high statistical efficiency and unbiased nature. The results were presented through scatter plots, forest plots, and a funnel plot. Finally, sensitivity analyses, including heterogeneity, horizontal pleiotropy, and leave-one-out (LOO) sensitivity tests, were conducted to ascertain the reliability of the MR findings (Supplementary Figure S1).

## 3 Results

### 3.1 Positive causal relationships between SFAs and the onset of RA

**Figure 1:**
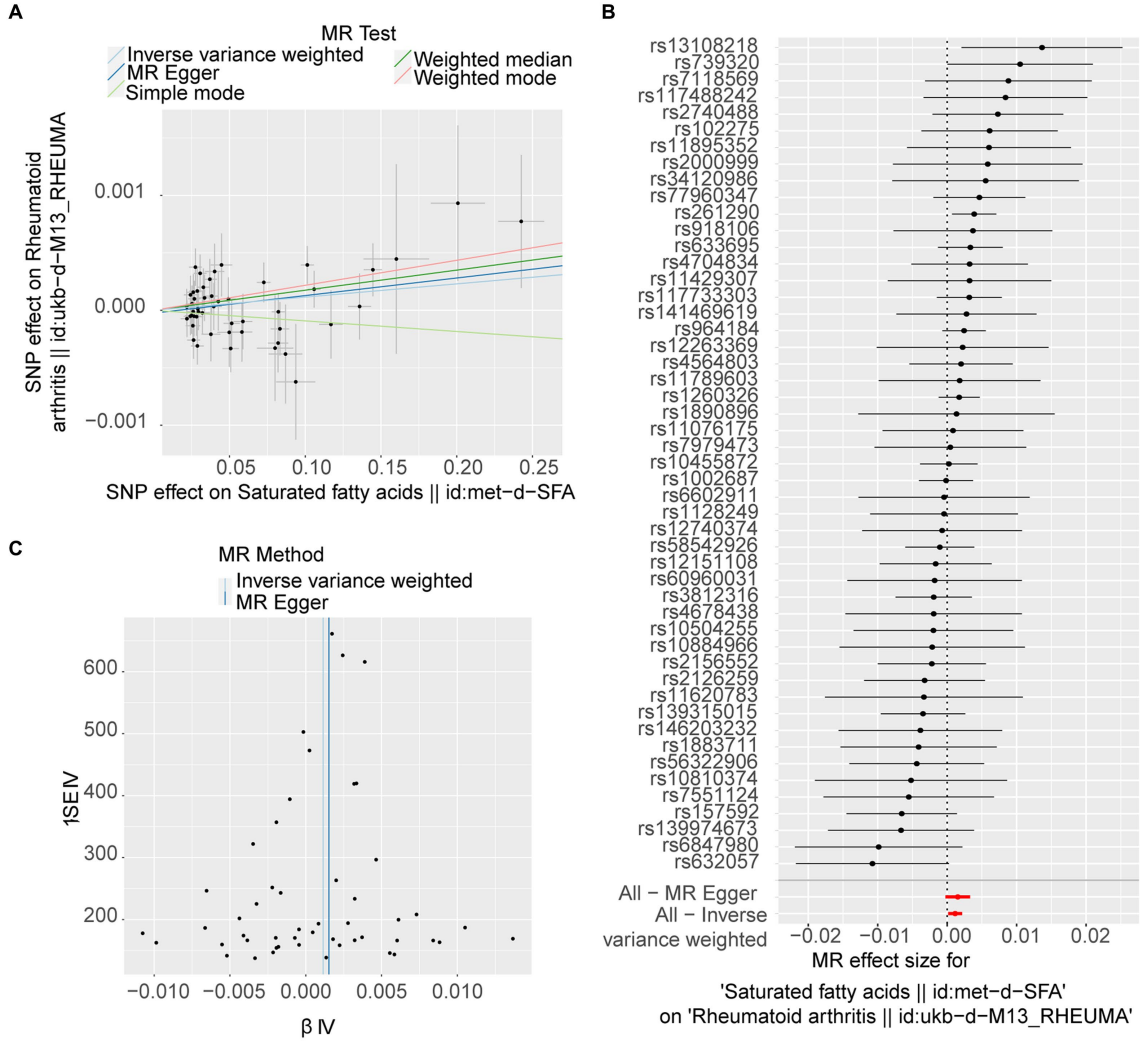
Forward Mendelian randomisation (MR) analysis of the causal effect of saturated fatty acids (SFAs) on rheumatoid arthritis (RA) occurrence. **(A)** The scatter plot of MR analysis. The X-axes show the SNP-exposure effect, and the Y-axes show the SNP-outcome effect. The positive slope reflects a positive causal effect of SFAs on RA. **(B)** Forest map of MR analysis, combining a Wald ratio method for each SNP effect (horizontal black solid line) and an inverse-variance weighted (IVW) method for fixed-effects (horizontal red solid line). The solid line completely on the right side of 0 indicates positive links between SFAs and the risk of RA. **(C)** Funnel plot of MR analysis. The SNPs are symmetrically distributed along both sides of the IVW line, indicating MR links to Mendel’s second law.

Following filtration, 52 independent SNPs of SFAs were identified as IVs (Supplementary Table S1). The forward MR results are presented in Table 1. A causal relationship was established between SFAs and RA (*p* = 0.025), with SFAs identified as a risk factor for RA (odds ratio [OR] = 1.001) according to the IVW method. The results of two other methods, weighted median and weighted mode, also supported this result (*p* < 0.05, OR > 1). The scatter plot indicated a minimal impact of confounding factors on the credibility of the forward MR analysis due to a negligible intercept, suggesting SFAs as a risk factor for RA with a positive slope based on the IVW method (Figure 1A). A forest plot was created to evaluate the diagnostic efficiency of each SNP of SFA for RA. Whilst the effects of individual SNPs of SFAs on RA were not significant, their collective impact in the IVW model was considerable, implying that SFAs are a potential risk factor for RA (Figure 1B). In addition, the forward MR analysis of the causal effect of SFAs on RA aligned with Mendel’s second law of random grouping (Figure 1C).

**Table 1:** Forward MR results of SFAs on the risk of RA.

Outcome	Exposure	Method	nSnp	pval	OR	OR_lci95	OR_uci95
RA	SFA	MR Egger	50	0.098	1.002	1.000	1.003
RA	SFA	Weighted median	50	0.027	1.002	1.000	1.003
RA	SFA	Inverse-variance weighted	50	0.025	1.001	1.000	1.002
RA	SFA	Simple mode	50	0.566	0.999	0.996	1.002
RA	SFA	Weighted mode	50	0.015	1.002	1.000	1.004

### 3.2 Sensitivity analysis illustrated the reliability of the forward MR results

**Figure 2:**
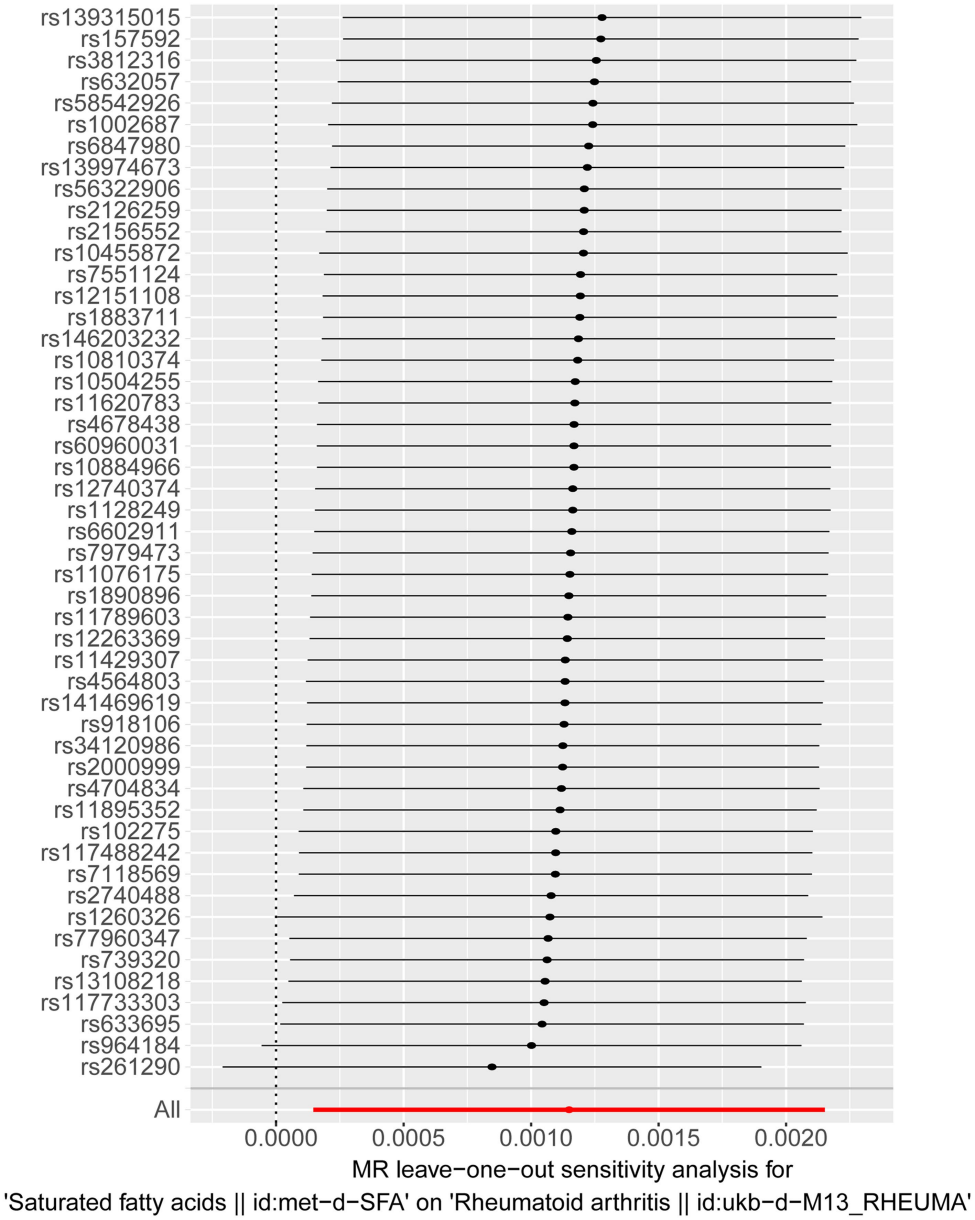
Results of a “leave-one-out” sensitivity analysis performed for SFAs on RA in the forward MR analysis. Calculate the MR results of the remaining SNPs after removing them one by one.

The sensitivity analysis further confirmed the reliability of the forward MR findings. There was no significant heterogeneity (Q_pval = 0.496) (Table 2) and no evidence of horizontal pleiotropy (*p* = 0.613) in relation to SFAs (Table 3). The LOO method was used to evaluate the influence of each SNP on the MR result, and the results revealed no instances of severe bias (Figure 2). Thus, SFAs were affirmed as a risk factor for the onset of RA with verified dependability.

**Table 2:** Sensitivity analysis based on the heterogeneity test.

Exposure	Outcome	Method	Q	Q_df	Q_pval
Saturated fatty acids || id:met-d-SFA	Rheumatoid arthritis || id:ukb-d-M13_RHEUMA	MR Egger	48.182	48	0.465
Saturated fatty acids || id:met-d-SFA	Rheumatoid arthritis || id:ukb-d-M13_RHEUMA	Inverse-variance weighted	48.441	49	0.496

**Table 3:** Sensitivity analysis based on the horizontal pleiotropy test.

Exposure	Outcome	Egger_intercept	se	pval
Saturated fatty acids || id:met-d-SFA	Rheumatoid arthritis || id:ukb-d-M13_RHEUMA	−2.49E-05	4.89E-05	0.613

### 3.3 No causal effect of RA on SFA

**Figure 3:**
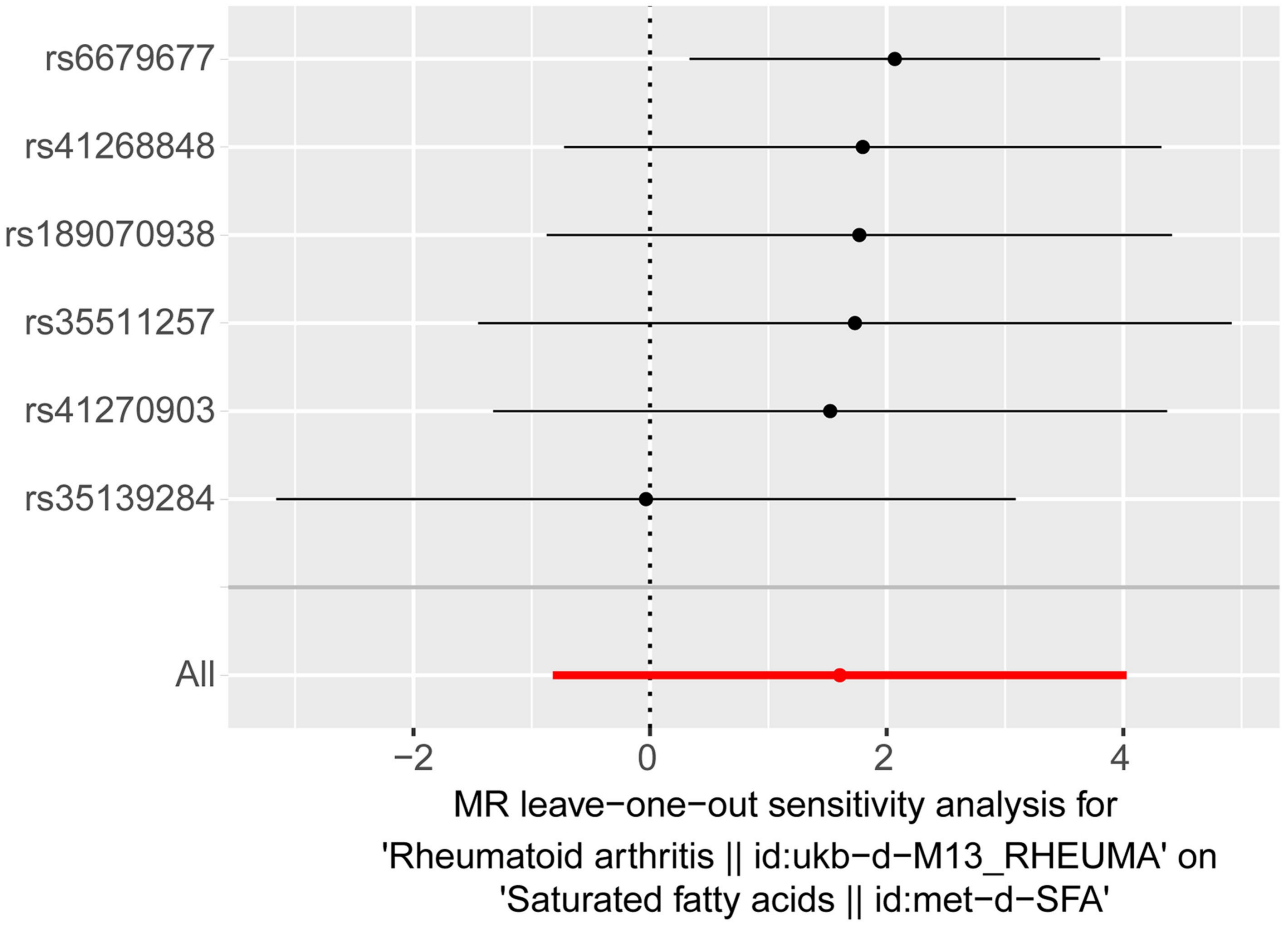
Results of a “leave-one-out” sensitivity analysis performed for RA on SFAs in the reverse MR analysis.

For the reverse MR analysis, seven independent SNPs were selected as IVs following filtration (Supplementary Table S2). The reverse MR results are presented in Table 4. The *p*-values for SFAs as an outcome were greater than 0.05, indicating no statistical significance (*p* = 0.195). This suggests the absence of a causal relationship between RA as the exposure factor and SFAs as the outcome. Although heterogeneity existed (Q_pval = 0.035), it did not impact the IVW results, meaning that the conclusion of the reverse MR analysis was reliable. Additionally, no horizontal pleiotropy (*p* = 0.201) or points of severe bias were detected (Figure 3). In summary, no causal effect of RA on SFAs was found.

**Table 4:** Reverse MR results of RA on the risk of SFAs.

Outcome	Exposure	Method	nSnp	pval	OR	OR_lci95	OR_uci95
SFA	RA	MR Egger	7	0.123	391.071	0.970	157671.258
SFA	RA	Weighted median	7	0.010	12.057	1.829	79.477
SFA	RA	Inverse-variance weighted	7	0.195	4.971	0.440	56.137
SFA	RA	Simple mode	7	0.392	4.137	0.212	80.852
SFA	RA	Weighted mode	7	0.038	11.393	2.085	62.268

## 4 Discussion

The exact aetiology and underlying pathogenic mechanisms of RA are not fully understood. Dietary components, as potential environmental factors, might influence early autoimmune responses in individuals who are genetically susceptible to RA. These effects are mediated by regulating the intestinal microbiota composition and function, altering gut permeability, and inducing immunomodulation (24, 25). Beyond conventional pharmacological approaches, dietary intervention is emerging as a promising complementary therapy for RA management. This study evaluated GWAS data in a bidirectional two-sample MR framework, providing solid genetic evidence of a causal link between SFAs and the risk of RA. Importantly, our analysis confirmed a positive causal association between these factors.

The current body of research supports the link between SFAs and RA. Serum or plasma is often used to study the relationship between fatty acids and health or disease states (26). Analysing serum or plasma can reveal the fatty acid status, which can serve as an indicator of health status and disease risk in a clinical setting (27). Notably, elevated levels of total SFAs and hexadecanoic acid (C16:0) have been observed in the plasma/serum and synovial fluid of patients with RA relative to controls (28–30). A comprehensive cross-sectional analysis from the National Health and Nutrition Examination Survey, including 16,530 participants and 1,053 patients with RA, highlighted a significant correlation between C16:0 and an elevated risk of RA, along with increased levels of hypersensitive C-reactive protein (31). Similarly, a prospective cohort study by Sebe et al. focussing on 53 female patients with RA showed a strong association between SFA intake and a greater than 5% decrease in the skeletal muscle index over 1 year; similar results were also observed in murine RA models (17).

However, the relationship between SFAs and RA continues to be a topic of debate. Using gas chromatography–mass spectrometry, Yao et al. found that lower levels of acetic and propionic acids in patients with RA were correlated with a higher percentage of regulatory B cells in peripheral blood, suggesting an immunomodulatory effect via free fatty acid receptor 2 (32). Additionally, the microbiota-derived short-chain fatty acid (SCFA) butyrate is believed to induce the differentiation of B cells towards a regulatory phenotype, which may in turn reduce arthritis severity through increasing the serotonin-derived metabolite 5-hydroxyindole-3-acetic acid (33, 34). Because of the inconclusive nature of these observational studies, which are often affected by factors such as age, drug usage, and lifestyle, this study utilised MR as an IV to establish a causal link between SFA exposure and RA outcome. The inherent random distribution of genetic variations helps to minimise confounding factors and reverse causality, thereby reinforcing the causal evidence (20, 35). Further sensitivity analyses were conducted using various methods to eliminate bias from both correlated and uncorrelated pleiotropy. Consequently, this study provides preliminary evidence of a causal relationship between SFA and RA, enhancing our understanding of the aetiological and risk factors involved in RA progression.

The specific biochemical pathways through which SFAs increase the risk of RA are not fully understood, although current studies suggest a link between inflammation and gut dysbiosis (12, 36, 37). Diets high in SFAs are shown to promote T cell activation and their differentiation towards Th1 and Th17 cells (38, 39). Concurrently, elevated expression of inflammatory mediators such as C-X-C motif chemokine receptor 3 intensifies the inflammatory response (38, 39). Zhou et al. revealed that hexadecanoic acid (C16:0) activates the STAT5-PI3K/Akt signalling pathway in T cells, leading to a marked upregulation of signal lymphocyte-activating molecule family member 3 and proinflammatory cytokines such as tumour necrosis factor-α, interleukin (IL)-1β, IL − 2, and IL-6 (40). Moreover, SCFAs, the primary metabolic products of gut microorganisms, influence gut microbiota composition, alter intestinal microbial metabolites, maintain intestinal mucosal integrity, and regulate immune homeostasis (41). For example, *Prevotella histicola* in the gut has been shown to delay arthritis onset in murine models by increasing the expression of butyrate and normalising the composition of the gut microbiota (42). Additionally, SFAs in the intestine have been implicated in promoting IL-22 production by inhibiting G protein receptor 41 and histone deacetylase (43).

Nevertheless, this study has several limitations. First, MR cannot pinpoint the exact biological mechanisms underlying the causal link between SFAs and RA, highlighting the need for further research. Second, the lack of data on specific SFA subtypes in databases such as the GWAS database limits the ability to identify the SFAs that should be reduced or eliminated to manage RA. Third, the unavailability of information on lifestyle, age, and other factors precludes subgroup analyses and the investigation of inter-subgroup differences in the effects of SFAs on RA. This study merely underscores that the data available on this topic are limited, and more comprehensive studies are urgently required.

In conclusion, this study confirmed a causal relationship between SFAs and the onset of RA, identifying SFAs as a significant risk factor for RA. Our findings suggest that elevated SFA levels may increase the risk of RA. Interventions aimed at modifying SFA levels could help to reduce the burden of this chronic disease. This research offers new insights into the genetic links between SFAs and RA, setting the stage for future investigations to explore the underlying mechanisms and inform early intervention strategies for RA.

## Data availability statement

The original contributions presented in the study are included in the article/Supplementary material, further inquiries can be directed to the corresponding author.

## Author contributions

XiY: Conceptualization, Methodology, Validation, Writing – original draft. YY: Formal analysis, Methodology, Validation, Writing – review & editing. ZJ: Data curation, Software, Validation, Writing – review & editing. WM: Funding acquisition, Investigation, Project administration, Resources, Supervision, Visualization, Writing – review & editing. XuY: Formal analysis, Writing – review & editing.

## Footnotes

^1^ https://gwas.mrcieu.ac.uk/

^2^ https://www.ukbiobank.ac.uk
