# Supplementary material for: The causal impact of saturated fatty acids on rheumatoid arthritis: a bidirectional Mendelian randomisation study
Source: Front Nutr. 2024 Feb 12;11:1337256. doi: 10.3389/fnut.2024.1337256 (PMC10895023; doi:10.3389/fnut.2024.1337256)
Supplement: Supplementary file 3 [file Data_Sheet_1.PDF]

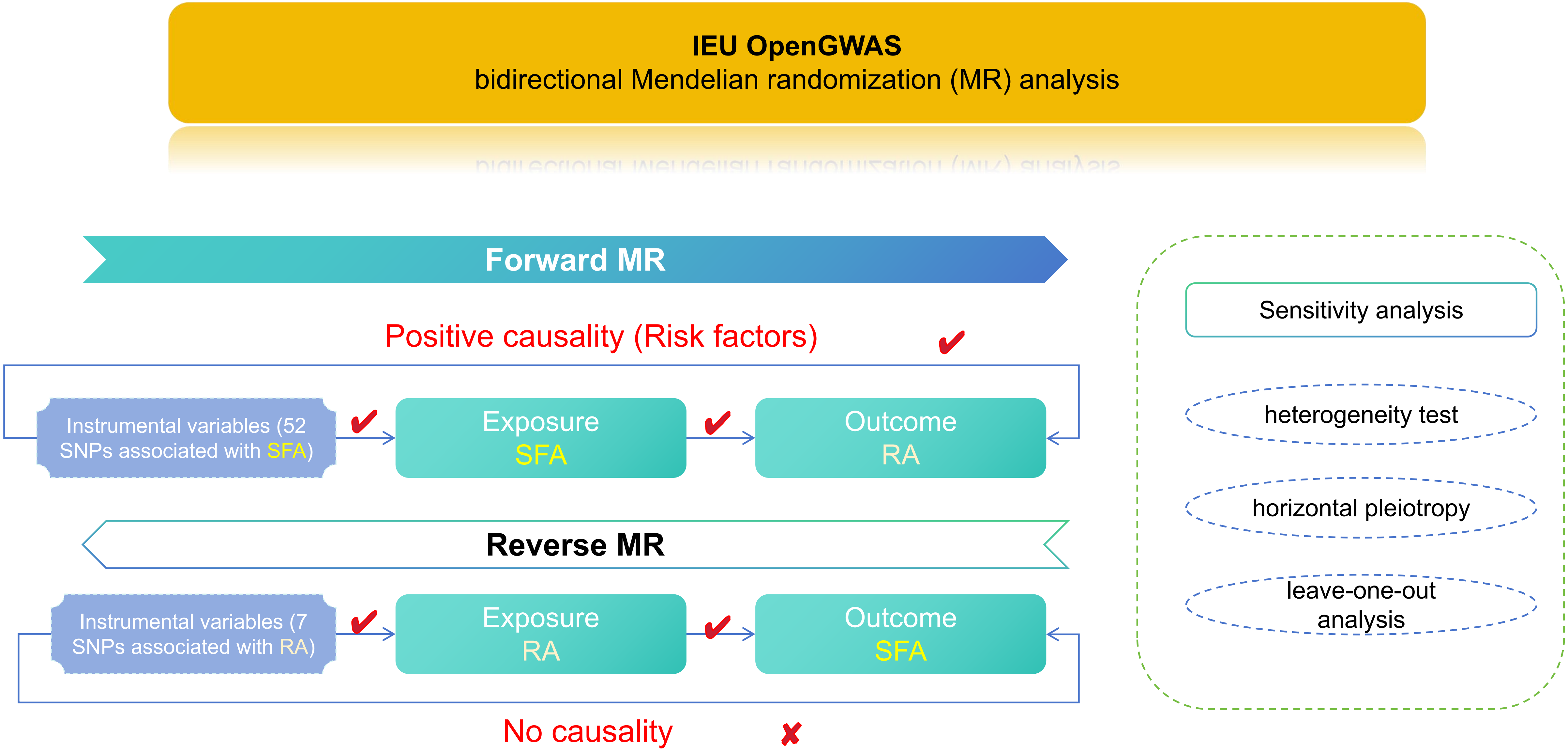
IEU OpenGWAS
bidirectional Mendelian randomization (MR) analysis
Forward MR
Positive causality (Risk factors)
Instrumental variables (52 SNPs associated with SFA)
Exposure
SFA
Outcome
RA
Reverse MR
Instrumental variables (7 SNPs associated with RA)
Exposure
RA
Outcome
SFA
No causality
Sensitivity analysis
heterogeneity test
horizontal pleiotropy
leave-one-out analysis
